# Induction of Proinflammatory Multiple Sclerosis-Associated Retrovirus Envelope Protein by Human Herpesvirus-6A and CD46 Receptor Engagement

**DOI:** 10.3389/fimmu.2018.02803

**Published:** 2018-12-06

**Authors:** Benjamin Charvet, Josephine M. Reynaud, Geraldine Gourru-Lesimple, Hervé Perron, Patrice N. Marche, Branka Horvat

**Affiliations:** ^1^International Centre for Infectiology Research, INSERM U1111, CNRS UMR5308, Ecole Normale Supérieure de Lyon, University of Lyon, Lyon, France; ^2^GeNeuro Innovation, Lyon, France; ^3^Institute for Advanced Biosciences, INSERM U1209, CNRS UMR5309, Université Grenoble-Alpes, IAPC, La Tronche, France

**Keywords:** human endogenous retrovirus, multiple sclerosis, MSRV, inflammation, HHV-6A, CD46, TLR4

## Abstract

The aberrant expression of human endogenous retrovirus (HERV) elements of the HERV-W family has been associated with different diseases, including multiple sclerosis (MS). In particular, the expression of the envelope protein (Env) from the multiple sclerosis-associated retrovirus (MSRV), a member of HERV-W family and known for its potent proinflammatory activity, is repeatedly detected in the brain lesions and blood of MS patients. Furthermore, human herpesvirus 6 (HHV-6) infection has long been suspected to play a role in the pathogenesis of MS and neuroinflammation. We show here that both HHV-6A and stimulation of its receptor, transmembrane glycoprotein CD46, induce the expression of MSRV-Env. The engagement of extracellular domains SCR3 and SCR4 of CD46-Cyt1 isoform was required for MSRV-env transactivation, limiting thus the MSRV-Env induction to the CD46 ligands binding these domains, including C3b component of complement, specific monoclonal antibodies, and both infectious and UV-inactivated HHV-6A, but neither HHV-6B nor measles virus vaccine strain. Induction of MSRV-Env required CD46 Cyt-1 singling and was abolished by the inhibitors of protein kinase C. Finally, both membrane-expressed and secreted MSRV-Env trigger TLR4 signaling, displaying thus a proinflammatory potential, characteristic for this viral protein. These data expand the specter of HHV-6A effects in the modulation of the immune response and support the hypothesis that cross-talks between exogenous and endogenous viruses may contribute to inflammatory diseases and participate in neuroinflammation. Furthermore, they reveal a new function of CD46, known as an inhibitor of complement activation and receptor for several pathogens, in transactivation of HERV *env* genes, which may play an important role in the pathogenesis of inflammatory diseases.

## Introduction

Human endogenous retrovirus (HERV) sequences are assumed to be remnants of ancient retroviral infections of our ancestral germ-line cells and constitute ~8% of the human genome (1). Although these viral sequences were generally silenced through diverse evolutionary mechanisms, some HERV genes have been shown to be expressed in controlled tissue-specific manner (2). Interestingly, some HERV were suggested to be involved in the pathogenesis of several autoimmune diseases, including multiple sclerosis (MS) (3–5) and type 1 diabetes (6), neuropathological syndromes like schizophrenia and bipolar disorder (3), as well as degenerative diseases, including amyotrophic lateral sclerosis (7, 8).

Two members of the HERV-W family have been particularly studied with respect to MS: MS-associated retrovirus (MSRV), and the HERV-W fixed copy on chromosome7q. Both are known to possess complete envelope protein ORF, MSRV-*env* and *syncytin*-*1*, respectively, sharing 93% of sequence identity. Free MSRV virus-like particles were repeatedly isolated from leptomeningeal cells, B-lymphocytes and monocytes from MS patients (9–12). Moreover, MSRV was associated with MS by numerous clinical studies (13, 14), the expression of HERV-W *env* was increased in MS patients and correlated to poor prognosis (15).

The mechanisms responsible for the activation of HERV gene expression are poorly understood. Infections by some herpesviruses were shown to have transactivating effects on HERV genes. While HSV-1 infection could activate the transcription of HERV *gag, env*, and LTR sequences *in vitro* (16), EBV infection induces the expression of *env* genes from different HERV, including HERV-W (17, 18). In addition, human herpesvirus (HHV)-6A and HHV-6B were shown to activate the transcription of the Env protein of HERV-K18 (19, 20). Interestingly, both EBV and HHV-6 were also associated to MS pathogenesis (21, 22). However, a more direct link demonstrating the involvement of HHV-6 in MS pathogenesis is still missing.

HHV-6A uses CD46 as its entry receptor (23). CD46 is ubiquitously expressed type I transmembrane glycoprotein described initially as a complement regulatory protein, binding C3b and C4b and acting as a co-factor in their factor I-mediated proteolytic cleavage, preventing thus complement deposition on host tissue (24). Another physiological ligand of CD46, the Notch family member Jagged-1, plays a role in Th1 cell responses (25). Extracellular part of CD46 contains 4 short consensus repeats (SCR) and one Ser-Thr-Pro-rich (STP) domain close to the membrane and alternative splicing mechanisms lead to the expression of different isoforms of the CD46 protein, which can be placed into two groups according to their cytoplasmic tail, CD46-Cyt1 or CD46-Cyt2. Human lymphocytes are known as the main targets for HHV-6 infection, although several cell types from central nervous system (CNS), including astrocytes and oligodendrocytes, have been successfully infected by HHV-6A and, with lower efficiency, by HHV-6B, (26). In addition, several other human pathogens use CD46 as a receptor, including measles virus vaccine strain, adenovirus B and D, *Streptococci* and *Neisseriae* (27).

This study initially aimed to analyze the potential link between HHV-6A infection and expression of HERV-W. We have demonstrated that both HHV-6A and engagement of its receptor CD46 with several ligands induce MSRV-Env expression. We further identified the CD46-Cyt1 isoform to be responsible for this effect. Finally, we demonstrated the proinflammatory potential of HHV-6A through the induction of MSRV-Env which in turn activates TLR4 receptor. These results provide important information on the cross-talk between HHV-6A binding to its CD46 receptor and the transactivation of HERV-*env* genes leading to inflammation, which may play an important role in the pathogenesis of inflammatory diseases.

## Materials and Methods

### Cells

Astroglyoblastoma cell line U87-MG (U87) (ATCC®HTB-14^TM^) and neuroblastoma SH-SY5Y (ATCC®CRL2266 ^TM^) cells were cultured in Dubelco's Modified Eagles Medium (DMEM, Gibco^TM^), complemented with 10% heat inactivated Fetal Calf Serum (FCS), 1% glutamine, 1% penicillin/streptomycin. T-cell line HSB-2 (ATCC®CLL 120.1^TM^) was cultured in RPMI-1640 (Gibco^TM)^, 10% of FCS, 1% glutamine, and 1% penicillin/streptomycin. Peripheral blood mononuclear cells (PBMC) from healthy donors were obtained from the “Etablissement Français du Sang” of Lyon (France). PBMC were isolated by Ficoll separation from blood samples and cultured in RPMI-1640 medium completed with 10% of FCS, 1% glutamine, and 1% penicillin/streptomycin. Healthy donors signed a written Informed Consent Form, documented at the Centre for Blood Transfusion of Geneva, allowing the commercial use of their blood and blood components for medical research after definitive anonymization. Cord blood mononuclear cells (CBMC) were kindly provided by Dr M. Ducdodon after density gradient centrifugation of human cord blood and CD34^+^ cells depletion using immunomagnetic beads (CD34^+^ MicroBead Kit, Miltenyi Biotec, Bergisch-Gladbach, Germany), as described previously (28). Umbilical cord blood was obtained from healthy full-term newborns with written parental informed consent according to the guidelines of the medical and ethical committees of Hospices Civils de Lyon and of Agence de Biomédecine, Paris, France. Experiments using cord blood were approved by both committees and were performed in full compliance with French law.

### Virus

HHV-6A (GS strain) and HHV-6B (HST), both kindly provided by Dr L. Naesens (Belgium), were propagated respectively, in the HSB-2 and MOLT-3 cell lines. For virus production, cells were infected at a multiplicity of infection (MOI) of 0.05 for 1–2 h at 37°C. At maximum cytopathic effect, cells were centrifuged, resuspended in 10-fold lower volume of RPMI supplemented with 20% of FCS, and frozen at −80°C. After 3 thawing-freezing cycles, virus suspensions were then clarified by a succession of centrifugation (10 min, 1,500 rpm, 4°C) and final supernatant was loaded on a 20% sucrose layer. Viruses were pelleted by ultracentrifugation (2 h, 28,500 rpm, SW32Ti rotor) and resuspended in cold RPMI. Non-infected cell suspensions were processed in identical conditions and obtained product was used for “mock” infections. Virus titers were determined by immunofluorescence. HSB-2 cells were cultured in 96-well plates and infected in quadruplicate with serial dilutions of viral stock. After 5 days of culture, cells were harvested on 10-well slides, fixed in formalin 2.5%, stained with mouse anti-p41 primary antibody (Santa Cruz Biotechnology) and Alexa Fluor 488- conjugated goat anti-mouse secondary antibody (Molecular Probes). Titers were calculated in tissue culture infective dose 50 per ml (TCID50/ml) or in PFU/ml. Recombinant measles virus (MV) IC323, expressing vaccine strain Edmonsoton H and EGFP was kindly provided by T. Yanagi (Japan) (29, 30) and was propagated on Vero fibroblasts and harvested from infected cells when a strong cytopathic effect developed. All virus stocks were free of mycoplasma, as tested by MycoAlert test (Lonza) and were conserved at −80°C.

### Infections and Stimulations

Infection of HSB-2 cells, CBMC, PBMC, with HHV-6A was performed in suspension in the small volume for 1 h 30 at 37°C with an indicated MOI. Cells were then pelleted and resuspended in fresh medium for further culture. U87 and SH-SY5Y cells were seeded in 6-well plates, and infected with HHV-6A, HHV-6B or MV with indicated MOI. In some experiments HHV-6A was inactivated using ultraviolet light (254 nm), for 5 min on ice, placing the lamp 15 cm above the tube containing the virus. The efficiency of viral inactivation was assessed by titration of inactivated virus and was used if at least 98% of inactivation was reached.

U-87 cells were stimulated with several anti-CD46 Abs recognizing different CD46 SCR domains: anti-SCR1 Tra2.1 (31, 32), anti-SCR1 MC20.6 (33), anti-SCR3-4 GB24 (34), anti-SCR4 MEM-258 (Biolegend) or rabbit polyclonal anti-CD46 antibody (35) in concentrations indicated in figure legends. Stimulation with murine IgG1 immunoglobulin MOPC-21 (BD Bioscience) was used as a control. C3b was obtained as described previously (36, 37) and used as 50 mM. Cells were cultured on cover glass slides for 24h, culture media was removed and replaced by treatment for 5h before PBS wash and fixation in 2.5% formalin during 10 min at room temperature (RT) before immunofluorescence staining or flow cytometry.

### Flow Cytometry

Cells were stained for membrane expression of MSRV-Env using murine IgG1 anti-MSRV-Env mAb GN_mAb_Env01 (38), provided by GeNeuro. Initially, 5 × 10^5^ to 10^6^ cells/well were plated in 96 well plates and centrifuged 3 min, 1,500rpm, at 4°C for all washings, using buffer containing 2.5% PBS, 0.02% NaN_3_. Cells were than incubated 20 min, 4°C, with anti-MSRV-Env (2 μg/10^6^ cells), in wash buffer (in PBS with 1% BSA for 30 min). After subsequent 3 washes, cells were stained for 20 min at 4°C with secondary anti-mouse IgG F(ab')2 fragment (Alexa Fluor 647 conjugate) (Cell Signaling Technology). Cells were subsequently washed and viable cells were acquired on FACSCalibur 3C cytometer (BD Biosciences, Belgium) and FACS analysis was performed using CellQuestPro (BD Biosciences) followed by FlowJo (Tree Star Inc., USA) analysis.

### Immunofluorescence Analysis

Cells were cultured on cover glass slides in 6 wells plates. After fixation (formalin 2.5%, 5 min at RT) cells were washed several times in PBS, permeablized using triton X-100 0.5% for 10 min. Saturation was performed using PBS/4% horse serum (30 min at RT) before incubation with specific mouse anti-MSRV-Env IgG1 (GN_mAb_Env01, GeNeuro) (38), diluted to 2 μg/ml, in PBS/4% horse serum/0.5% Triton-X100, during 1 h at RT. In those experiments where murine mAbs were used to stimulate CD46, MSRV-Env was stained using GN_mAb_Env01 directly coupled with Alexa 555 dye using Zenon^TM^ Alexa fluor^TM^ 555 mouse IgG1 labeling kit (Molecular Probes). In some experiments, anti-MSRV-gag mAb (GN_mAb_Gag06 GeNeuro) or isotype control IgG1 were used in the same conditions. After multiple washes, cells were incubated with the secondary donkey anti-mouse antibody conjugated with Alexa 555 (Molecular Probes), diluted to 1/750 in PBS/4% horse serum/0.5% Triton-X100 (1 h, RT). HHV-6 staining was performed using biotinylated anti-HHV-6 p41 Ab (9A5D2, Santa Cruz) followed with streptavidin-Alexa 488 (ThermoFisher) staining. The nuclei were counterstained with DAPI (Sigma). After multiple washes in PBS, mounting was performed using Fluoroprep (BioMérieux). Cells were analyzed using an Axioplan 200 imaging microscope (Zeiss).

### Inhibition of CD46 Signaling Pathways

U87 cells were pretreated with small molecule inhibitors, dissolved in DMSO for 45 min, washed and then stimulated with HHV-6A (MOI = 1) for 5 h. Following inhibitors were used: 5 μM Bisindolylmaleimide I (Protein Kinases C inhibitor, Sigma) (39) or 5 μM Src Inhibitor-1 (Src kinase inhibitor, Sigma) or 40 μM Apigenin (Casein Kinase 2 inhibitor, Sigma) (40).

### Inhibition of CD46-Cyt1 Expression by si-RNA

Control siRNA and CD46 Cyt1-specific siRNA (AUACCUAACUGAUGAGACCUU) were obtained from Dharmacon (Perbio, France), and used as previously described (41). U87 cells (5 × 10^4^ cells) were cultured in 6-well plates for 1 day in OptiMEM (Invitrogen), supplemented with 10% FCS before transfection of 30 nM siRNA with Lipofectamine RNAiMAX (Invitrogen) according to manufacturer's instructions. mRNA expression level was assessed by quantitative RT-PCR after 2 days, as described below. Results are presented as means ± standard errors of the means (SEM) (*n* = 3).

### Quantitative RT-PCR

RNA was isolated from cells using a NucleoSpin RNA extraction kit (Macherey-Nagel) following the manufacturer's instructions. Total RNA quantification and integrity was assessed using Experion RNA StdSens analysis kit (Bio-Rad, CA). Next, cDNA was generated from 1 μg of total RNA using iScript reverse transcriptase (Bio-Rad). TaqMan probes and primers specific for human MSRV *env*, decribed to distinguish it from HERV-W7q *env* syn 1 (42) were used: MSRV env: F: CTTCCAGAATTGAAGCTGTAAAGC, R: GGGTTGTGCAGTTGAGATTTCC, probe: FAM-5′-TTCTTCAAATGGAGCCCCAGATGC AG-3′TAMRA In addition, the following primers were used: GAPDH: F: AGCAATGCCTCCTGCACCACCAAC, R: CCGGAGGGGCCATCCACAGTCT, tbp: F: GCGGTTTGC TGC GGT AAT CAT, R: GAC TGT TCT TCA CTC TTG GCT CCT GT [as described: 20)], all purchased from Eurogentec. Quantification of transcripts was performed with the CFX96 Real-time PCR detection system (Bio-Rad) and all reactions were done in 10 μl mixtures using iQ SYBR Green Supermix or TaqMan iQ Supermix according to the manufacturer's instructions (Bio-Rad). Data were analyzed with the Bio-Rad CFX Manager Software. The reactions were optimized to reach an efficiency of 2 so that a relative ΔΔCt quantification approach could be used (43). Each sample was normalized to the endogenous control gene (GAPDH and tbp), and the transcript which did not change significantly following the infection in the given cell type was chosen for the final quantification. All data are presented as the fold change relative to the control.

To determine the expression of CD46 Cyt1 and Cyt2 isoforms, reverse transcription was performed on 0.5 μg of total RNA using oligo(dT) and random-hexamer oligonucleotide primers (iScript cDNA synthesis kit; Bio-Rad), amplified on a Biometra Tpersonal PCR device (Biometra), and cDNAs were diluted 1/10. Quantitative PCR was performed using Cyt1 and Cyt2 primers described in Astier et al. (44): Cyt1 forward: CTAACTGATGAGACCCACAGAGAAGT, reverse: TCAGCTCCACCATCTGCTTTC, Cyt2 forward: GAAGAAAGGGAAAGCAGATGGT, reverse: CCTCTCTGCTCTGCTGGAGTG. GAPDH was used as housekeeping gene, amplified by primers as described above. Results are means ± standard errors of the means (SEM) (*n* = 3). Experiments were performed in order to validate the interpretation of quantitative real-time PCR (RT-qPCR) data following the MIQE guidelines (45).

### TLR4 Signaling Assay

HEK-Blue™-hTLR4 cells (Invivogen) were used to study the stimulation of human TLR4 by monitoring the activation of NF-kB, as described by supplier's instructions. Cell line was obtained by co-transfection of the human or murine TLR4, MD-2, and CD14 co-receptor genes, and an inducible SEAP (secreted embryonic alkaline phosphatase) reporter gene into HEK293 cells. The SEAP reporter gene is placed under the control of an Il-12 p40 minimal promoter fused to five NF-κB and AP-1 binding sites. Stimulation with a TLR4 agonist activates NF-κB and AP-1, which induce the production of SEAP, assessed by a colorimetric assay. HEK-Blue™-hTLR4 culture was performed according to supplier's recommendations. In initial experiments, stimulation by LPS (LPS-EK ultrapure, Invivogen) was performed to test the system efficiency. HEK-Blue™-hTLR4 cells were cultured in presence of HHV-6A, supernatant of uninfected or HHV-6A infected U87 cells (MOI: 0.1; 24 h post infection). In addition, the co-culture of HEK-Blue™-hTLR4 and uninfected or HHV-6A infected U87 cells (MOI: 0.1; 24 hpi) were performed to promote cell-cell contacts. All stimulations were performed during 16 h and TLR4 activation was revealed colorimetrically with QUANTI-Blue™ detection assay (Invivogen). Optical densities were measured with a TECAN infinite-200 plate reader (TECAN) at 620 nm. Results were expressed in corrected DO (DO of untreated condition were subtracted from all other conditions) and obtained from 3 independent experiments.

### Statistical Analysis

Student's *t*-test was used to compare each group to the control group when data passed the normality test, otherwise Mann–Whitney rank sum test was used to compare non-normal data. *P*-values < 0.05 were considered significant. Statistical analyses were performed with Prism 5.04 (GraphPad Software) and used to plot the data and for calculations.

## Results

### HHV-6A Infection Induces the Expression of MSRV-*env*

**Figure 1:**
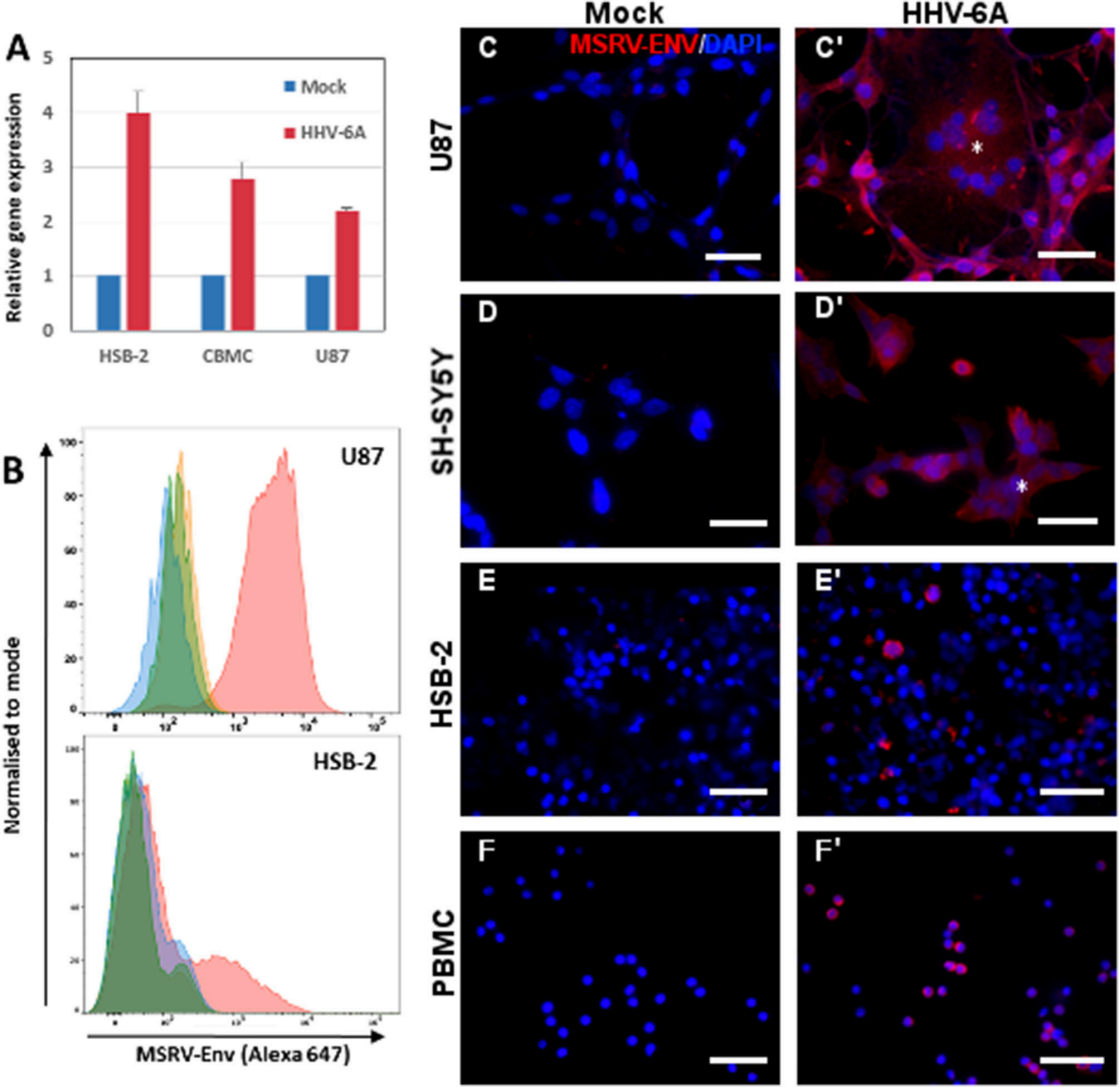
HHV-6A infection induces expression of MSRV-Env in different cell types. **(A)** HSB2 cells, cord blood mononuclear cells (CBMC) and U87 cells were infected with HHV-6A at MOI 1 or incubated with the mock control for 24 h and MSRV *env* expression was analyzed by RT-qPCR. The values are expressed relatively to those in mock-infected cells and error bars represent SEM of 3 independent experiments. **(B)** Cytofluorometric analysis of U87 and HSB-2 cells infected or not with HHV-6A for 24 h at MOI 0.1 and stained by anti-MSRV-Env mAb, followed by anti-mouse Ig-Alexa 647 (green: non-infected cells + secondary Ab staining; orange: non-infected cells + complete staining; blue: infected cells + secondary Ab staining, pink: infected cells + complete staining). **(C**,**C')** U87, **(D**,**D')** SH-SY5Y, **(E,E')** HSB-2, and **(F,F')** peripheral blood mononuclear cells (PBMC) were either incubated with mock preparation **(C–F)** or infected at MOI 0.1 **(C'–F')** and analyzed 24 h later by immunofluorescence using anti-MSRV-Env mAb, followed by anti-mouse Ig-Alexa 555 (red staining). HHV-6A induced syncytia formation of adherent infected cells **(C',D'**, *). DAPI (blue staining) was used to visualize cell nuclei (Bar = 50 μm). Data are representative of at least three independent experiments.

**Figure 2:**
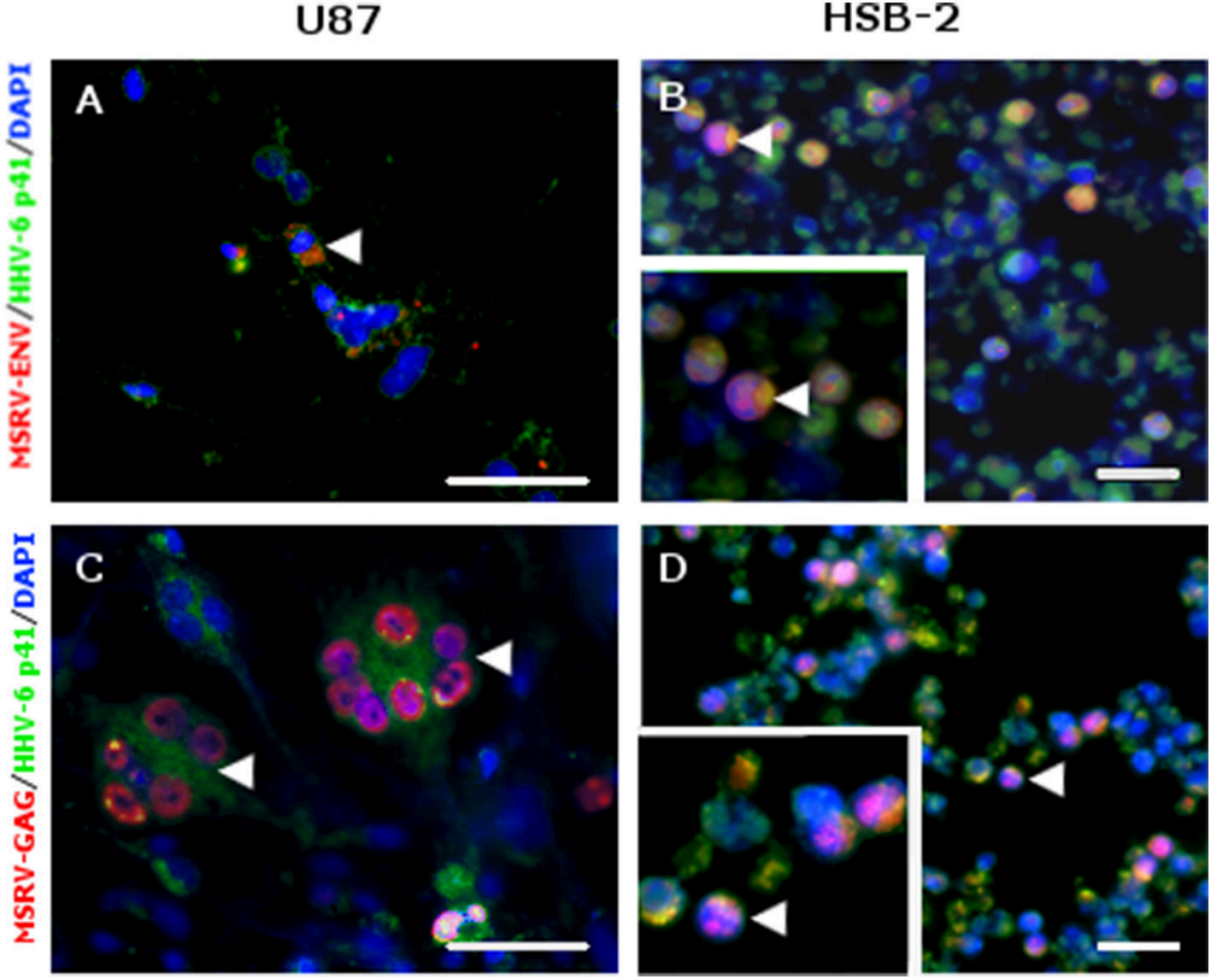
MSRV-ENV and MSRV-GAG were expressed in HHV-6A infected cells. **(A,C)** U87 and **(B,D)** HSB-2 cells were infected with HHV-6A during 24 h. Cells were fixed and stained for MSRV-ENV (GN_mAb_Env01) **(A,B)** or MSRV-GAG (GN_mAb_Gag06) **(C,D)** followed by anti-mouse-Alexa 555 (red staining). HHV-6 staining was revealed using biotinylated anti-HHV-6-p41 mAb, followed by streptavidin-FITC (green staining). HHV-6A infected cells expressing either MSRV-ENV or MSRV-GAG were pointed by arrowhead. DAPI (blue staining) was used to visualize cell nuclei. Bar = 50 μm. Bottom left frames **(B,D)**: higher magnification of cell pointed by arrowhead.

To analyze the capacity of HHV-6A infection to transactivate MSRV-*env* gene, we examined initially the level of mRNA expression of MSRV-*env* in few cell types susceptible to HHV-6A infection: T-cell line HSB-2, cord blood mononuclear cells (expected to be naive for HHV-6A infection) and astroglyoblastoma cell line U87-MG (U87). We observed the induction of MSRV-*env* transcription in both primary cells and cell lines (Figure 1A), suggesting that either HHV-6A infection or contact with the viral particles may awake MSRV-*env* transcription. Then, we analyzed the induction of MSRV-Env at the protein level, following HHV-6A infection, using MSRV-Env-specific mAb GN_mAb_Env01 (38) (Figure S1). Cytofluorometric analysis revealed the strong surface expression of MSRV-Env in infected HSB-2 and U87 cells (Figure 1B). Further immunofluorescence analysis confirmed the expression of MSRV-*env* at the protein level in infected peripheral blood mononuclear cells (PBMC), as well as HSB-2 and neuroglial cell lines U87 and SH-SY5Y (Figures 1C–F). In the absence of any stimulation MSRV-Env expression was not detected in any of the tested cell types (Figure S2), confirming further the specificity of utilized mAb. HHV-6A infection of analyzed cell types was further confirmed by staining with anti-HHV-6A p41 Ab (Figures 2A,B and data not shown)

**Figure 3:**
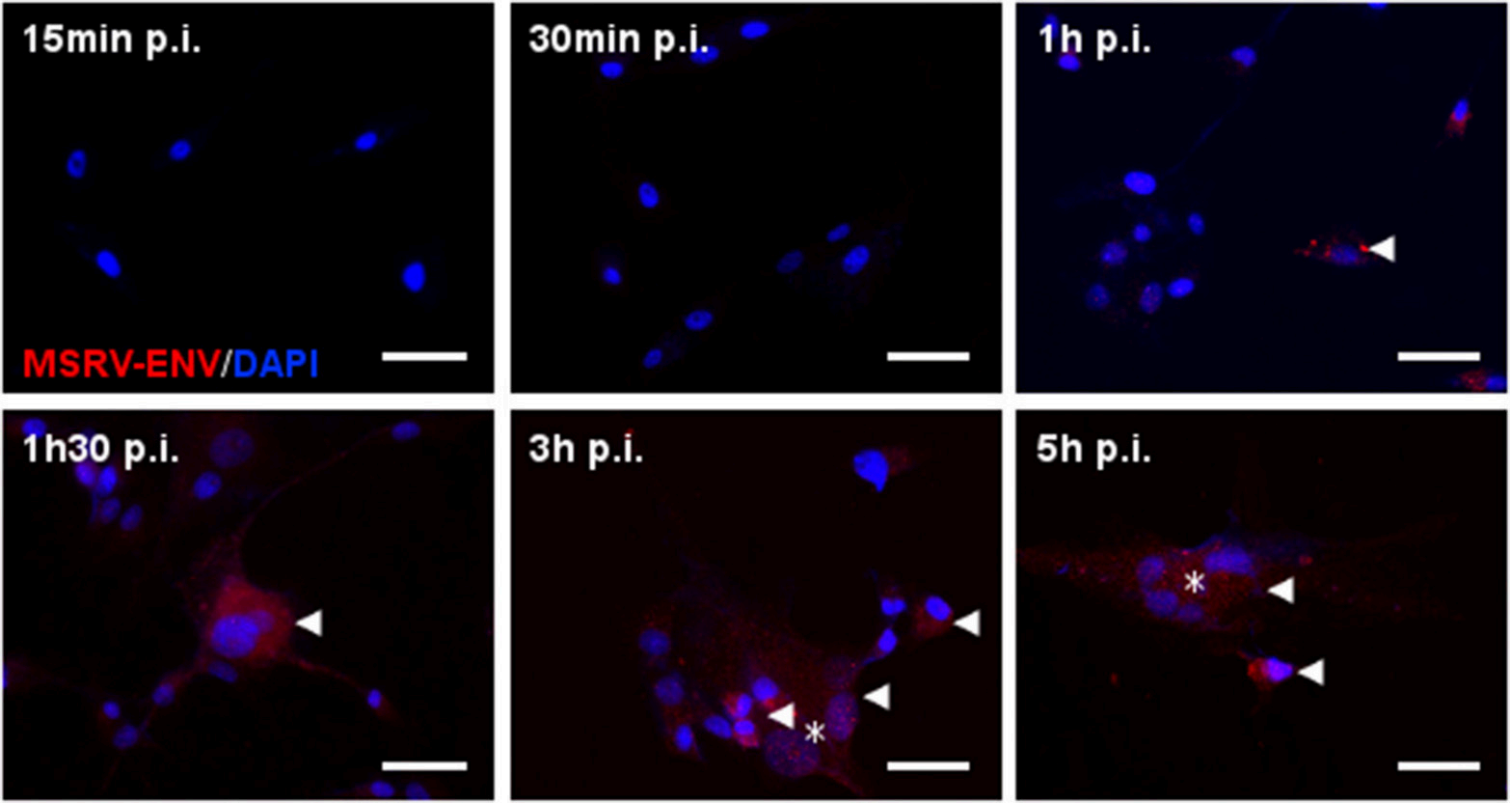
Kinetics of HHV-6A-induced MSRV-Env production in U87 cells. U87 cells were infected with HHV-6A (MOI 1) and analyzed at indicated time points after infection by immunofluorescence using anti-MSRV-Env mAb, followed by anti-mouse-Alexa 555 (red staining, arrowhead). HHV-6A induced syncytia formation observed at 3 h p.i. (*). DAPI (blue staining) was used to visualize cell nuclei. Bar = 50 μm.

In addition to MSRV-*Env* transactivation, HHV-6A infection induced also MSRV-gag expression (Figures 2C,D), suggesting its potential for the production of the complete MSRV particles. The kinetics of expression was further analyzed in U87 cells (Figure 3). The induction of MSRV-Env was observed rapidly, starting already 1 h after contact with the virus, suggesting that viral replication was not necessarily required.

### Engagement of CD46 Domains SCR3 and SCR4 Induces Expression of MSRV-Env

**Figure 4:**
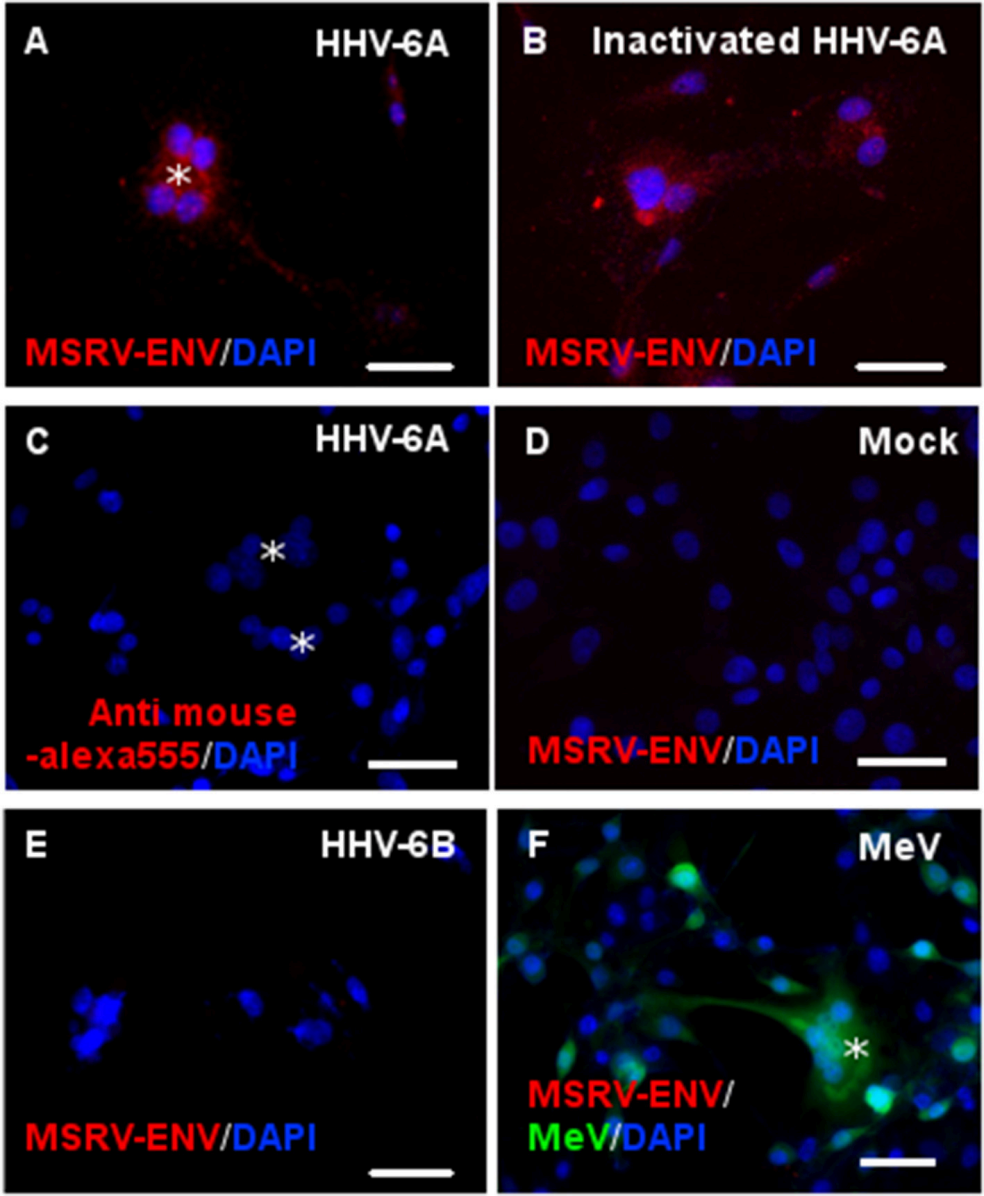
Both infectious and UV-inactivated HHV-6A, but neither HHV-6B nor Measles virus, could induce MSRV-Env expression. U87 cells were either infected or put in contact with UV-inactivated virus and observed 24 h later for the expression of MSRV-Env. Following viruses at MOI 1 were used: **(A,C)** HHV-6A, **(B)** UV-inactivated HHV-6A, **(D)** mock-infected control, **(E)** HHV-6B, and **(F)** recombinant measles virus (MeV) vaccine strain expressing EGFP (green staining, *syncytia formation). The expression of MSRV-Env was detected using anti-MSRV-Env mAb immunofluorescence analysis, followed by anti-mouse-Alexa 555 (red staining), including DAPI (blue staining) to visualize cell nuclei (bar = 50 μm). Alternatively, primary mAb was replaced with the isotype control **(C)**.

We further analyzed whether the stimulation of HHV-6A receptor CD46, by different ligands, could induce the MSRV-Env expression in U87 cells (Figure 4). While UV-inactivated HHV-6A induced MSRV-Env similarly to the infectious HHV-6A (Figures 4A,B), neither HHV-6B nor measles virus presented any detectable effect (Figures 4C,D). In contrast to HHV-6A, closely related HHV-6B is known to use CD134 as an entry receptor (46), which may prevent the induction of MSRV-Env *via* engagement of the viral receptor. Nevertheless, measles virus vaccine strain uses CD46, more particularly its domains SCR1 and 2 (32), but did not have any effect of MSRV-Env induction either (Figure 4D). As HHV-6A is known to bind distinct CD46 domains from measles virus, notably SCR 2 and 3 (47), we further analyzed the potential effect of the other CD46 ligands, binding different CD46 SCRs.

**Figure 5:**
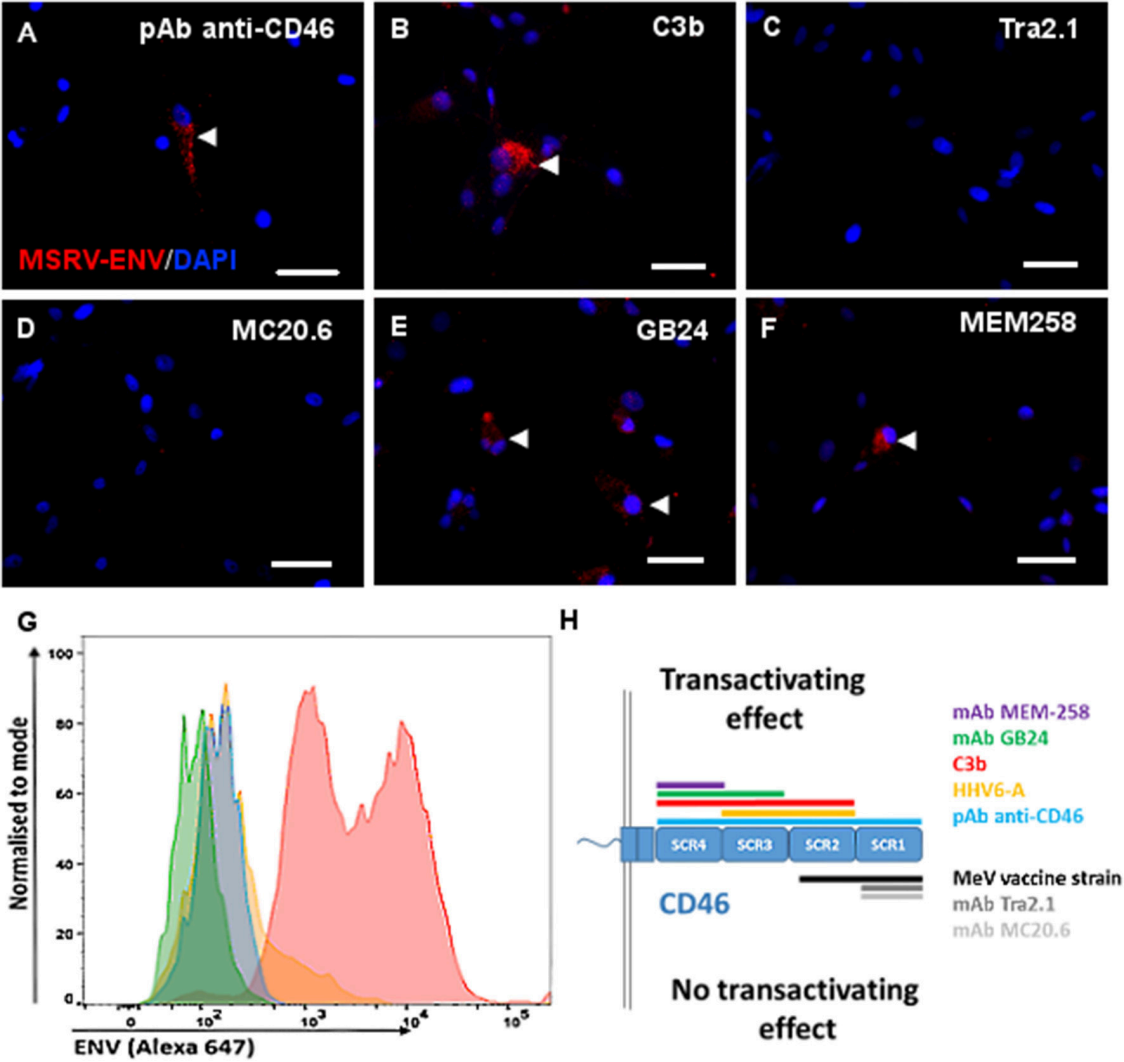
Engagement of CD46-SCR3 and/or CD46-SCR4 trigers intracellular and cell surface MSRV-Env expression. Several CD46 ligands known to bind different CD46 SCR domains were used to stimulate U87 cells: **(A,G)** anti-CD46 rabbit polyclonal antibody, **(B)** C3b component of complement (50 mM), **(C)** Anti-SCR1 Tra2.1 (75 μg/ml), **(D)** anti-SCR1 MC20.6 (75 μg/ml), **(E)** anti-SCR3-4 GB24 (75 μg/ml), **(F)** anti-SCR4 MEM-258 (50μg/ml). The expression of MSRV-Env was detected using anti-MSRV-Env mAb-coupled to Alexa 555 by immunofluorescence analysis (red staining, arrowhead), including DAPI (blue staining) to visualize cell nuclei (bar = 50 μm) **(A–F)**, or by cytofluorometry **(G)**, using anti-MSRV-Env mAb followed with anti-mouse-Alexa 647 (**blue**: cells stimulated with anti-CD46 + secondary Ab staining, **pink**: cells stimulated with anti-CD46 followed by complete anti-MSRV-Env staining, **green**: cells stimulated with isotype control mAb + staining with secondary Ab; **orange**: cells stimulated with isotype control mAb followed by complete anti-MSRV-Env staining). Good cell viability was observed in all stimulation conditions. **(H)** Schematic representation of CD46 SCR domains recognized with utilized CD46 ligands, having a different effect on the induction of MSRV-Env.

Interestingly, C3b component of complement, known to bind to CD46 SCR domains 2-4 (48, 49) induced MSRV-Env expression (Figure 5B), confirming the viral infection is not mandatory and suggesting the necessity for the engagement of particular domains of CD46 for the Env induction. To further analyze which domain has critical importance in the transactivation, we used a panel of anti-CD46 Abs, recognizing different regions of the extracellular CD46 (Figure 5). While polyclonal anti-CD46 Ab induced the expression of both intracellular and membrane MSRV-Env (Figures 5A,G), only mAbs GB24 and MEM258, recognizing either both SCR3 and 4 or only SCR4 respectively, were capable of triggering MSRV transactivation (Figures 5E,F). In contrast, mAbs MC20.6 and Tra2.1, recognizing SCR1, and used in the concentration known to block measles virus infection in our previous experiments (data not shown). did not affect MSRV-Env expression (Figures 5C,D). Altogether, these results strongly support the conclusion that the engagement of CD46 SCR3 and 4 is required for the induction of MSRV-Env expression (Figure 5H).

### CD46-Cyt1 Isoform Engagement Requires Protein Kinase C to Induce MSRV-Env Expression

**Figure 6:**
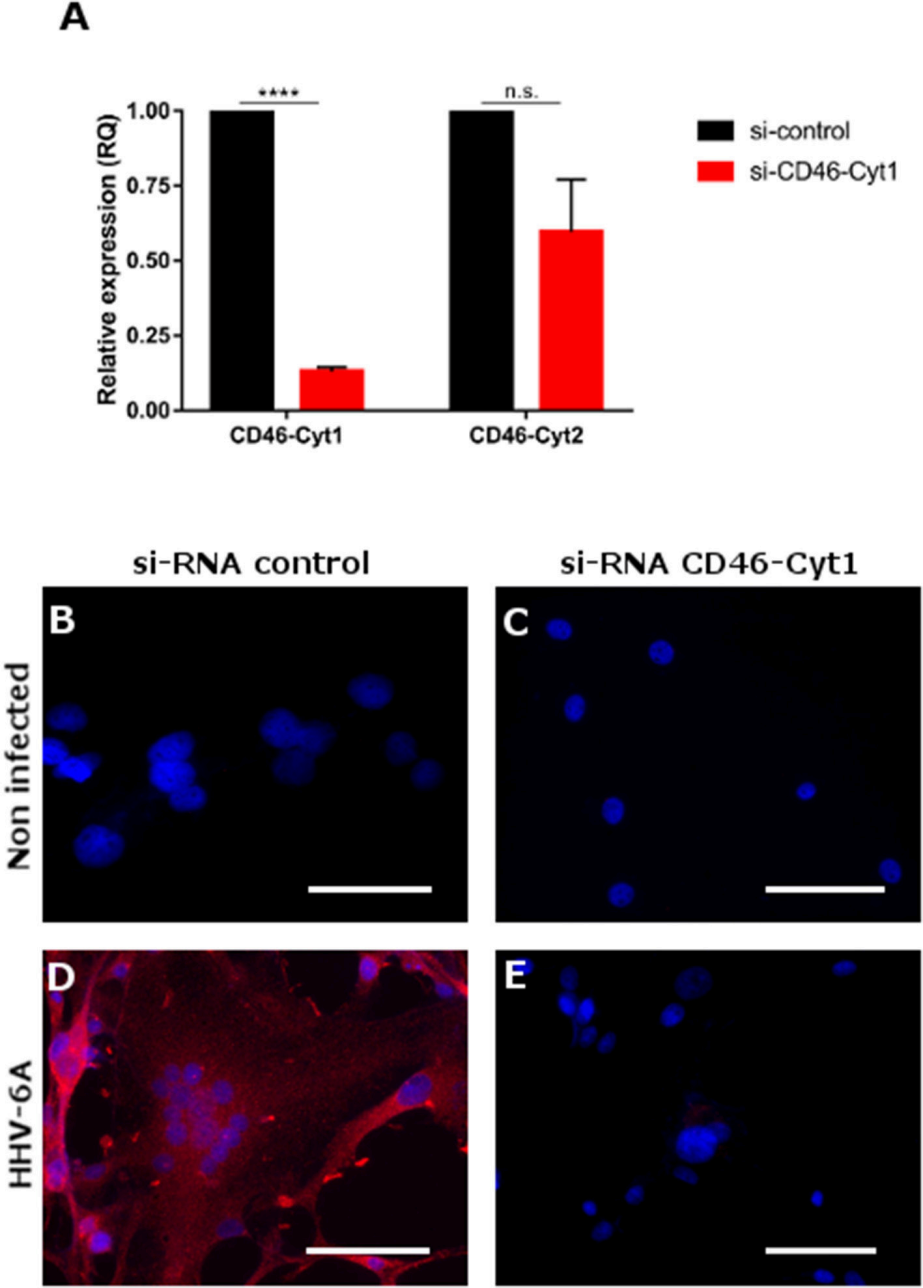
Induction of MSRV-Env expression is dependent of CD46-Cyt1 isoforme. **(A)** CD46-Cyt1 and CD46-Cyt2 mRNA expression levels in si-CD46-Cyt1-treated U87 cells normalized to si-control treated cells, analyzed by RT-qPCR (relative expression). Error bars, mean ± *SD* of three experiments, statistical test used: nonparametric KS test (n.s. *P* > 0.1, ****P < 0.0001, vs. untreated condition) **(B–E)** MSRV-Env expression in U87 cells, treated with either si-RNA control **(B,D)** or and si-Cyt1 **(C,E)**, and either left non-infected **(B,C)** or infected with HHV-6A (MOI 0.1) for 24 h **(D,E)**. MSRV-Env expression was determined using anti-MSRV-Env mAb, followed by anti-mouse-Alexa 555 (red staining) cellular nuclei were visualized using DAPI (blue staining), bar = 50 μm.

Due to the alternative splicing the CD46 protein could have two cytoplasmic tails, CD46-Cyt1 and 2, known to engage different signaling pathways (50). We thus analyzed which CD46 cytoplasmic isoform is involved in the induction of MSRV-Env expression (Figure 6). Inhibition of CD46-Cyt1 expression by si-RNA in U87 cells (Figure 6A) led to the loss of HHV-6A-induced MSRV-Env expression (Figures 6B–E), suggesting the critical role of this cytoplasmic domain in the transactivation pathway.

**Figure 7:**
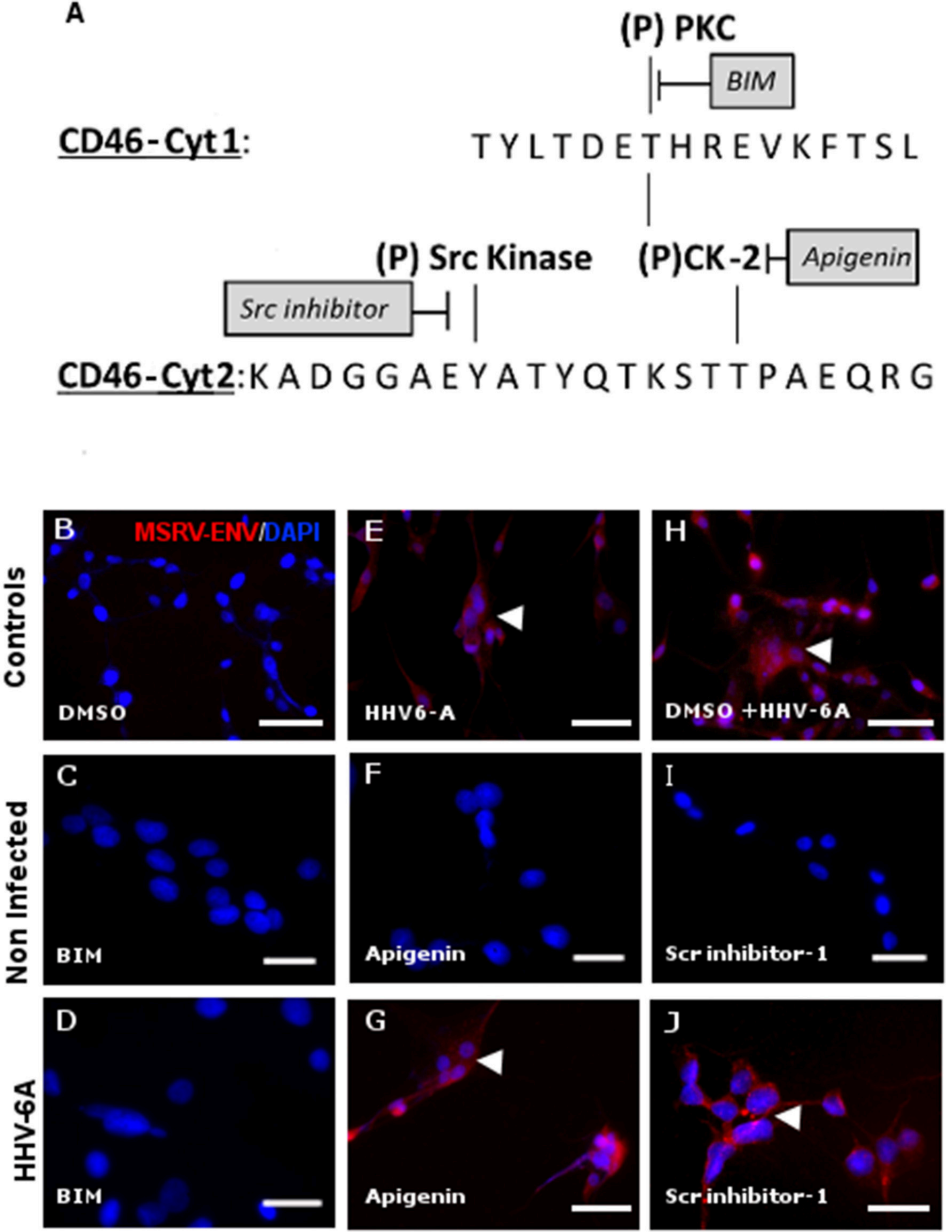
Activation of MSRV-Env expression requires protein kinase C activity. **(A)** Schematic representation of putative phosphorylation sites within CD46-Cyt1 and Cyt2 and candidate kinases and their inhibitors (PKC: Protein Kinases C; CK-2: Casein Kinase 2). **(B,E,H)** Transactivation controls. **(C,D)** Effect of bisindolylmaleimide 5 μM (BIM) on HHV-6A-induced MSRV-Env expression; **(F,G,I,J)** absence of the effect of, Apigenin 40 μM and Scr-inhibitor 5 μM on MSRV-Env expression. The expression of MSRV-Env was analyzed by immuofluorescence, using anti-MSRV Env mAb, followed by anti-mouse-Alexa 555 (red staining, arrowhead) and representative images from 3 independent experiments were presented. DAPI (blue staining) was used to visualize cell nuclei, bar = 50 μm.

CD46 is known to induce different intra-cellular signals, which can lead to diverse changes in cellular activities (51, 52). CD46-Cyt1 possess the putative signal for tyrosine phosphorylation by protein kinase C (PKC) and casein kinase 2 (CK-2), while CD46-Cyt2 has the presumed signal for tyrosine phosphorylation by src kinases and CK-2 (53, 54) (Figure 7A). We used specific inhibitors for each of these kinases and analyzed whether subsequent HHV-6A infection could still trigger MSRV-Env expression Figures 7B-J). Strikingly, highly selective, cell-permeable PKC inhibitor bisindolylmaleimide (BIM) completely abrogated MSRV-Env induction (Figure 7D), while the CK-2 inhibitor Apigenin and Src kinase inhibitor-1 did not exhibit any effect (Figures 7G,J). In agreement with the implication of CD46-Cyt1 in MSRV-Env induction seen in Figure 6, these results suggest the necessity of CD46-Cyt1 tyrosine phosphorylation and PKC function in the triggering MSRV-Env expression.

### Activation of the Human TLR4 by HHV-6A-Induced MSRV-Env

**Figure 8:**
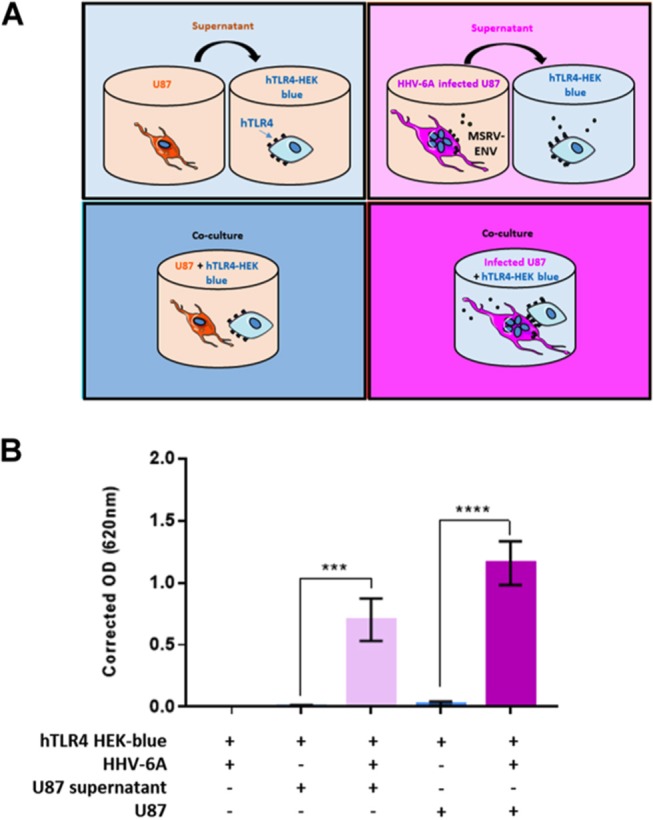
HHV-6A-induced MSRV-Env triggers human TLR4. **(A)** Schematic presentation of experimental conditions. HEK cells co-expressing human TLR4 (hTLR4) and TLR4 activation reporter gene (SEAP), were stimulated with either HHV-6A, supernatants from HHV-6A infected or non-infected U87 cells or co-cultured in presence of U87 cells previously infected with HHV-6A or not. hTLR4 activation was monitored colorimetrically using a SEAP reporter gene placed under the control of an NF-κB inducible promoter. **(B)** Data are expressed as corrected absorbance at 620 nm (absorbance obtained for HEK-blue + HHV-6A, mean: 0.27 ± 0.3, was subtracted from the absorbance obtained for other experimental conditions). Statistical analysis was performed using *t*-test on means ± SEM from three independent experiments (***P < 0.001, ****P < 0.0001, vs. uninfected conditions).

Recombinant MSRV-Env was shown to act via TLR4 to induce the inflammatory response in endothelial cells (55) and to inhibit oligodendroglial precursor cell differentiation (56). The extracellular part of MSRV-Env, but not the closely related HERV-W-Env syncytin-1, can trigger TLR4 signaling in primary human monocytes and dendritic cells (57). To determine whether HHV-6A-induced MSRV-Env may have a similar functional effect, we analyzed its capacity to stimulate human TLR4 receptor using as a functional assay HEK-Blue hTLR4 cells, where activation of TLR4 can be monitored through a SEAP reporter gene (58) (Figure 8A). Several experimental conditions were used, allowing to test the effect of MSRV-Env either expressed on the surface of HHV-6A-infected U87 cells or liberated in the supernatant. Indeed, HHV-6A infection of U87 cells strongly activated TLR4 receptor signaling, following either direct contact with HEK-Blue-hTLR4 indicator cells or after the exposure to the culture supernatant from infected U87 cells (Figure 8B). These results thus demonstrate that HHV-6A-induced both cell membrane-expressed and secreted MSRV-Env is functional and could exhibit a proinflammatory action *via* TLR4.

## Discussion

The regulation of the expression of HERV genes and their potential link to different diseases is just starting to be understood (16). Our study identifies CD46 as a critical factor in HHV-6A-mediated MSRV induction. Similarly, EBV was shown to transactivate HERV-K18 before the transcription of viral genes, by docking to the human complement receptor CD21 (59). Furthermore, HHV-6B strain PL, shown to require CD46-Cyt1 for infection (60), induced the expression of HERV-K18 mRNA in PBMCs without requirement for viral transcription and replication (20). Understanding the regulation of HERV-Env expression requires the differentiation whether a given factor is a cause or a result of particular disease or physiological condition. In that context, CD46 represents a connection between different host and environmental factors with the intracellular machinery as: (A) it binds C3b and C4b component of the complement and regulates their cleavage; (B) it also binds six different human pathogens [reviewed in Cattaneo 27)]; (C) it facilitates endosomal TLR9 triggering by DNA adenoviruses (61); (D) it promotes measles virus-derived peptide presentation by MHC-II molecules (62, 63); and (E) it was described to be an important link between innate and adaptive immunity (36). Therefore, CD46 presents remarkable cell membrane candidate which may allow the extracellular signals to “wake up” certain HERV genes and may thus answer to some of the key questions in the regulation of HERV-Env expression.

Although CD46 signaling pathways have been thoroughly studied in lymphocytes (50), they are largely unknown in neuroglial cells. CD46-Cyt2 tyrosine phosphorylation by src kinase Lck was previously observed in Jurkat T cell line (53). In addition, the infection of human epithelial cells by Neisseria, using CD46 SCR3 and STP domain as a receptor, leads to the Cyt2 tyrosine phosphorylation (64). Although CD46-Cyt1 is involved in intracellular signaling and interacts with several intracellular proteins, including scaffold proteins DLG (65) and GOPC (41) and serine-threonine kinase SPAK (66), this is the first demonstration of the potential role of Cyt1 phosphorylation by PKC-dependent pathway for signaling through CD46. Interestingly, the transactivation of HERV-K18 by EBV through its interaction with EBV cellular receptor CD21, known as complement receptor 2, was shown to be dependent on PKC activation as well (59), suggesting a common signaling pathway in the transactivation of HERV genes.

As MSRV-Env has a powerful immunopathogenic potential to activate an inflammatory cascade through interaction with TLR4 and has been closely associated to the pathogenesis of MS, our findings further underline the link between MS and different environmental factors including viral infection, such as HHV-6A. Indeed, many clinical studies have shown that HHV-6 DNA presence is detected more frequently in the blood of MS patients than in healthy donors (67–70), suggesting a putative association between HHV-6 infection and MS pathogenesis. Furthermore, MSRV transcripts and antigens have also been detected more frequently and in higher levels in the blood of MS patients (13, 14). HHV-6A-induced increase in MSRV-Env expression in human glial cell lines demonstrated here, supports the hypothesis that HHV-6 infection in MS patients could increase the expression of MSRV genes, which may in turn participate in the establishment of inflammation, via TLR4 stimulation, therefore promoting the immune disorder.

Our study demonstrated that stimulation with several CD46 ligands, including inactivated HHV-6A, C3b component of the complement and anti-CD46 antibodies, up-regulates the MSRV-Env expression. This strongly suggests that *env* transactivation could be triggered through a direct interaction between CD46 and its ligands, such as bacteria's using CD46 SCR3 and 4 as a receptor, in addition to HHV-6A, revealing new outcome of CD46 stimulation. It may thus contribute to a feedback-loop maintaining a stimulation, after the initial pathogen-induced activation. Indeed, CD46 is known to trigger different intra-cellular signals which can lead to diverse changes in cellular activities (50). CD46-induced MSRV-*env* production may be involved in the generation of the inflammatory process by pathogens using CD46 as a receptor, but also in other conditions where production of C3b component of the complement is induced. This would contribute to the multifactorial etiology of different human diseases, including MS. Strikingly, the alterations of CD46 regulatory functions in T-lymphocytes as well as in dendritic cells have been observed in patients with MS [reviewed in Astier 71)]. In this context, we could hypothesize that CD46 pathway could be dysfunctional in certain patients, as it has be shown in some chronic inflammatory diseases (50, 72), and if such alterations occur in glial cells, they could facilitate MSRV transactivation in the CNS. Further understanding the role of CD46 in the induction of HERV-Env should provide an important insight in the field and help identifying novel targets for preventive and therapeutic interventions.

## Author Contributions

BH, PM, BC, and JR designed the research. BC, JR, and GG-L performed research. HP contributed to research follow-up, manuscript review and the elaboration of accurate conditions for experiments with HERV reagents.

### Conflict of Interest Statement

PM is inventor of a patent on anti-Env inhibitory Ab owned by INSERM and HP receives compensation for his work by GeNeuro and is inventor on patents owned by BioMérieux, INSERM, or GeNeuro, but has transferred all his rights to BioMérieux or to GeNeuro under applicable laws for employed inventors. The remaining authors declare that the research was conducted in the absence of any commercial or financial relationships that could be construed as a potential conflict of interest.
