# Supplementary material for: Induction of Proinflammatory Multiple Sclerosis-Associated Retrovirus Envelope Protein by Human Herpesvirus-6A and CD46 Receptor Engagement
Source: Front Immunol. 2018 Dec 6;9:2803. doi: 10.3389/fimmu.2018.02803 (PMC6291489; doi:10.3389/fimmu.2018.02803)
Supplement: Supplementary file 1 [file Data_Sheet_1.docx]

**SUPPLEMENTARY FIGURES**

**
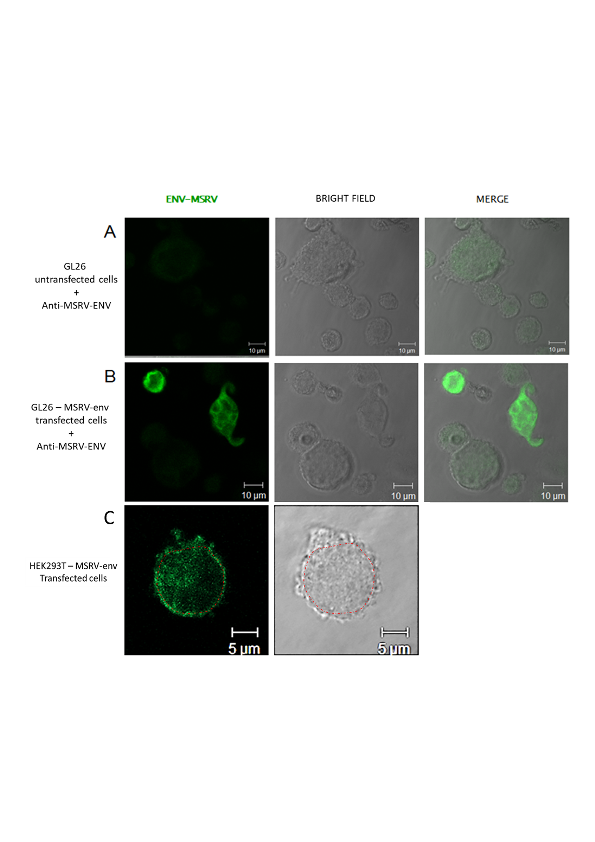
**

**Figure S1.** Anti-MSRV-Env mAb recognizes MSRV-Env expressed in transfected cells. Human glioblastoma cell line GL26 and embryonal kidney cell line HEK 293T were transfected with HERV-W-*env* and analyzed by confocal microscopy for the expression of MSRV-Env, using Z-stack projection (thickness 10µm). Anti-MSRV-Env mAb stained transfected GL26 and HEK239T cells (**A, C**) but not nontransfected GL26 cells (**A**). Single confocal slice of transfected cell (thickness 1µm) allows to observe an enrichment of MSRV-ENV at cell membrane (highlighted by the dotted red line) (**C**).


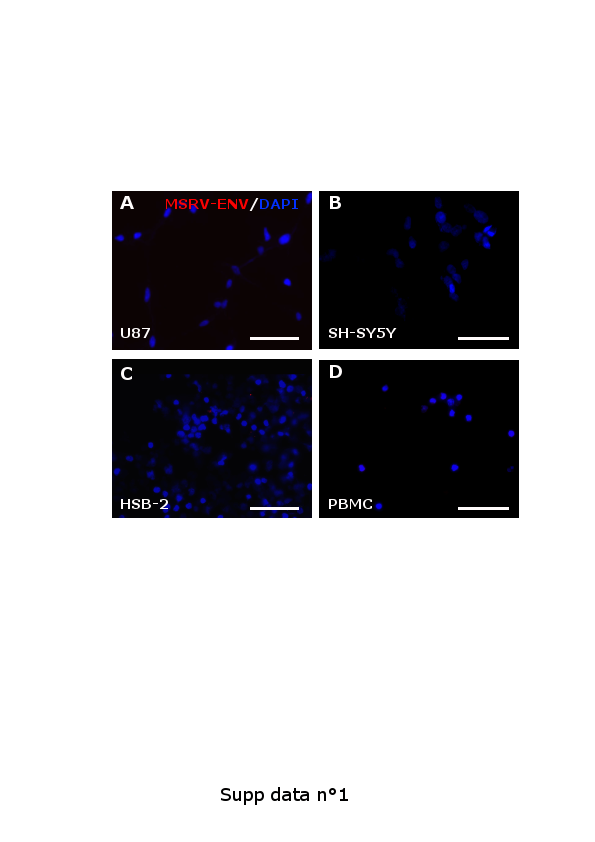


**Figure S2.** MSRV-ENV was not expressed in unstimulated cells. Cells in the absence of infection and any particular stimulation: U87 (A), SH-SY5Y (B), HSB-2 (C) cell lines and PBMCs (D) were stained using anti-MSRV-Env mAb (GN_mAb_Env01, followed with anti-mouse-Alexa 555 (red staining). DAPI (blue staining) was used to visualize cell nuclei. Bar = 50µm.
